# Supplementary material for: Hyper-Production of Pullulan by a Novel Fungus of Aureobasidium melanogenum ZH27 through Batch Fermentation
Source: Int J Mol Sci. 2023 Dec 25;25(1):319. doi: 10.3390/ijms25010319 (PMC10779298; doi:10.3390/ijms25010319)
Supplement: Supplementary file 1 [file ijms-25-00319-s001.zip › ijms-2636291-supplementary.pdf]

Supplementary Data

Hyper-production of pullulan by a novel fungus of *Aureobasidium melanogenum* ZH27 through batch fermentation

Qin-Qing Wang[a,b,†,*], Jia Lin[a,†], Qian-Zhi Zhou[a], Juan Peng[a], Qi Zhang[a], and Jiang-Hai Wang[a,*]

[a] Guangdong Provincial Key Laboratory of Marine Resources and Coastal Engineering, School of Marine Sciences, Sun Yat-Sen University, Zhuhai, 519082, China; qinqingw@163.com (Q.-Q. W.); linjia26@mail.sysu.edu.cn (L.J.); zhouqzh6@mail.sysu.edu.cn (Q.-Z. Z.); pengj28@mail.sysu.edu.cn (J.P.); zhangq528@mail2.sysu.edu.cn (Q.Z.); wangjhai@mail.sysu.edu.cn (J.-H. W.)

[b] Guangdong Engineering Laboratory of Biomass High-Value Utilization, Institute of Biological and Medical Engineering, Guangdong Academy of Sciences, Guangzhou, 510316, China; qinqingw@163.com (Q.-Q. W.)

* Correspondence: qinqingw@163.com (Q.-Q. W.); wangjhai@mail.sysu.edu.cn (J.-H. W.)

† These authors have contributed equally to this work.

Supplementary Figures

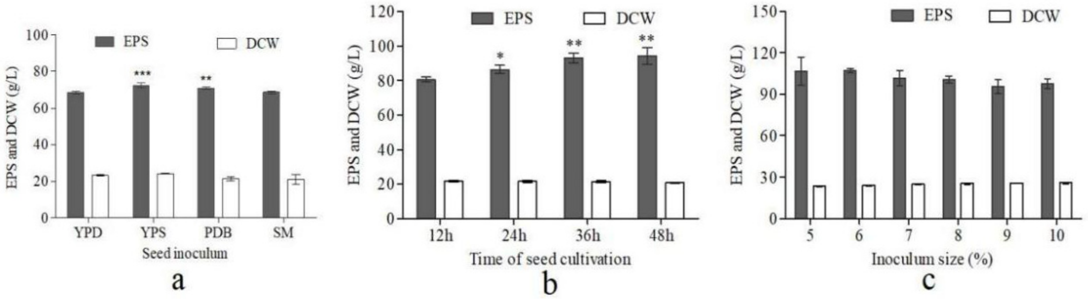

Figure S1. Selection and optimization of seeds for producing pullulan by ZH27.
a, seed inoculum; b: time of seed cultivation; and c: inoculum size.

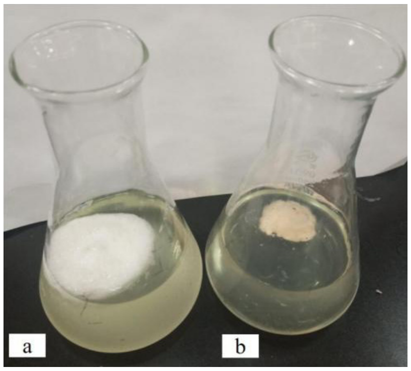

Figure S2. Pullulan appeared in the fermented supernatants of strain ZH27 cultivating under OC (a) and IC (b) at 132 h, respectively.

Supplementary Tables

Table S1. Quantitative real-time PCR primers of the genes involved in syntheses of pullulan, FOSs, trehalose, and glycerol.

Gene name	Primer sequence (5'→3')
β-actin	F: CGGTGTCACTCACGTTGTCC
	R: TCGAGAGCGACGTAGCAGAG
GluT1	F: CATCTCTGCCGTCAACGCC
	R: GAGCCAGTTGGAGGCGGTAG
GluT2	F: TGCGAGTGGATCGTTGGTATC
	R: TGGAACGGATGGGAAGAGG
GluT3	F: CGCTGTTCAGTTCGCTTGG
	R: CGTATTCGTGGTTGGCGGT
GluT4	F: CGAAGCTGTCGCTGCATCT
	R: GTTGCCTCCGAGCAGTTGT

GluT5	F: CATCGCTATGCTCATCGTCGG<br>R: GCGCCTACGAGCTTTCCTCTT
SUC2	F: TCTACCTCAGATACGACGCGC<br>R:GCAGGAGGTCTGGGTGTAAAG
GluK	F: ACTACCACGCTACCATCCGCA<br>R: CAGCATCGGCAATGTCAAAGC
PGM	F: GGTGGTGACGGTCGTTACTGG<br>R: GATACCACCAGTGGCCTTGC
UGP	F: TGGAGATCATCCCCAACGG<br>R: TGACGGGAAGGAAACGACG
UGT1	F: TTGCAACGAACATACCCTGGAC<br>R: AGAGCGACACCAAGACCATAAG
CreA	F: ACCTGCCGCGTCCTTACAAGT<br>R: CTCATCGGAACGACTGAAGCG
Apo	F: ACTGGTGTCGGTGCCTCGTA<br>R: CCCATCTGCGAGTGTGCCT
Gltp	F: GCAACACCCTCAGCAAGCAC<br>R: TTCTCGAGGCTGCTCAACC
PUL1	F: GTTGATCATGGTTGGGAAGGC<br>R: AGAGCGAAGATGGAGAAGCG
α-amylase gene	F: ACCTCTGGCAGCATCAGCAAT<br>R: TGGCCTTGGTAGATGATTGGG
Glucoamylase gene	F: AGACTGTCAACAACCCCAGCG<br>R: GTAGGCAATCATGGCAGTCGC
Pullulanase gene	F: GCAACGTCCTGGTGCAGAAAG<br>R: GACCTGGATTCGAGCCATTGC
Ags2	F: TCATTGCCGTCATCGTTCAGA<br>R: AGATGGGCAAGAACCAGGAGTG
Msn2	F: CCTCAAAAGCAGCAGCACATC<br>R: TGATGGTAGGCTTCTGGTGTTG
PFK26[a]	F: GTGTTGTTATGGTCGGTCTGC<br>R: AGGATTGGGAGTGGTGGTAC
FBA1	F: TGGTGGTGAGGAGGATGGTGT<br>R: TTCTGGTGCTTGGAGAGGAGG
G3P	F: AACATCCCCTGGGCTGAGAC<br>R: GGACCTGGATGTCGGACTTGT
PGK	F: AGCTCGACTACTTCGCTCAGG<br>R: AAAGCCATACCACCGCAGAC
PYK	F: GACAAGGCCGATCTCCGTTTC<br>R: TTGACACCCTGCTGGTTCTCG
PYD	F: CTACACGCAGCAAAGAACCCC<br>R: CCATCACCAGCATACACACCG
TPS1	F: CATCAAGGGTGTTCCTCAGAAGC<br>R: TTTGCCGTTGATTCTGCCG
TPS2	F: CACATCAACCCCTGGGATCTG<br>R: GTCCATGTCTGCACAGTGTTGGT
GPP	F: TACCAGGCAGCCGATCTATCTT<br>R: GCCCATCACCTCCAACCAT
GPD	F: CGCATACGACCCTCCTACCAT<br>R: TCGGTAGGCAGAGGGTAAAGC
Hog1	F: CCTGGTCAACGAGAACTGCG<br>R: GCTTGCCCTCGAGCATCTC
Sho1	F: CGCAACGTGATCGATTCCT<br>R: GTTGCTGTCCATTGCTCCCA
FF1	F: GGCAACTTCTCTGGCAACGAT<br>R: GTGTCTTTCTGCCTGATTCGG
FF2	F: TGTTCAGTGGCTCAGCAGTGG<br>R: ATCACGGAACTGAGTCGAGCC
FF3	F: CAATCAAGCATTCACCGTCCC<br>R: ACTCGCTTCCGACAACATGCT
FTR1	F: TATGCCATCCAGCGTCACCG<br>R: GCAGCCAACACGAGCCTTGTT
AreA	F: CGGAAGTCCAAAGCCTGGTGAT<br>R: TCTTCAAACTCAGCGGACGCAC

AreB	F: CTCGTGCAAAGGAAGCATCAG<br>R: ACTCAGACGGAGACGCTTAGGT

[a]Cited from Jiang et al. (2018)[1].

Table S2. Characteristic absorption peaks in the purified EPS and commercial pullulan.

Assignments	Wave number (cm^{-1})	
	**Commercial pullulan**	**Purified EPS**
–OH	3,396.03	3,423.03
C–H	2,925.48	2,923.60
O–C–O	1,641.13	1,637.29
C–O–H	1,419.35<br>1,371.14	1,427.09<br>1,371.16
C–O–C	1,157.08	1,159.03
C–O	1,022.09	1,025.96
α-1,6-glucosidic linkage	931.45	933.38
α-D-glucopyranose	848.53	852.40
α-1,4-glucosidic linkage	761.74	755.97

Table S3. Comparison of pH values, pullulan titers and viscosities of supernatants at the end of fermentation of strain ZH27 under IC and OC.

Medium	Final pH value	Supernatant viscosity (mP·s)	Pullulan titer (g/L)
Initial medium	2.97 ± 0.06	10753 ± 652.8	48.8 ± 3.8
Optimized medium	3.57 ± 0.06**	306 ± 8.54**	115.4 ± 1.8**

Reference

1. Jiang, H.; Xue, S.-J.; Li, Y.-F.; Liu, G.-L.; Chi, Z.-M.; Hu, Z.; Chi, Z. Efficient transformation of sucrose into high pullulan concentrations by *Aureobasidium melanogenum* TN1-2 isolated from a natural honey. *Food Chem* **2018,** 257, 29-35.
